# Ecological niche modelling for predicting the risk of cutaneous leishmaniasis in the Neotropical moist forest biome

**DOI:** 10.1371/journal.pntd.0007629

**Published:** 2019-08-14

**Authors:** Agathe Chavy, Alessandra Ferreira Dales Nava, Sergio Luiz Bessa Luz, Juan David Ramírez, Giovanny Herrera, Thiago Vasconcelos dos Santos, Marine Ginouves, Magalie Demar, Ghislaine Prévot, Jean-François Guégan, Benoît de Thoisy

**Affiliations:** 1 Laboratoire des Interactions Virus-Hôtes, Institut Pasteur de la Guyane, Cayenne, French Guiana; 2 Laboratoire des Ecosystèmes Amazoniens et Pathologie Tropicale, EA3593, Medicine Department, Université de Guyane, Cayenne, French Guiana; 3 Laboratório de Ecologia de Doenças Transmissíveis na Amazônia, EDTA Instituto Lêonidas e Maria Deane, FIOCRUZ, Amazonas, Brazil; 4 Grupo de Investigaciones Microbiológicas-UR (GIMUR), Programa de Biología, Facultad de Ciencias Naturales y Matemáticas, Universidad del Rosario, Bogotá, Colombia; 5 Parasitology Unit, Instituto Evandro Chagas (Secretaria de Vigilância em Saúde, Ministério da Saúde), Ananindeua, Brazil; 6 Laboratoire Associé du CNR Leishmaniose, Laboratoire Hospitalo-Universitaire de Parasitologie-Mycologie, Centre Hospitalier Andrée Rosemon, Cayenne, French Guiana; 7 Unité Mixte de Recherche MIVEGEC, Université de Montpellier, IRD, CNRS, Montpellier, France; 8 Unité Mixte de Recherche ASTRE Cirad-INRA, Université de Montpellier, Montpellier, France; Universidade do Estado do Rio de Janeiro, BRAZIL

## Abstract

A major challenge of eco-epidemiology is to determine which factors promote the transmission of infectious diseases and to establish risk maps that can be used by public health authorities. The geographic predictions resulting from ecological niche modelling have been widely used for modelling the future dispersion of vectors based on the occurrence records and the potential prevalence of the disease. The establishment of risk maps for disease systems with complex cycles such as cutaneous leishmaniasis (CL) can be very challenging due to the many inference networks between large sets of host and vector species, with considerable heterogeneity in disease patterns in space and time. One novelty in the present study is the use of human CL cases to predict the risk of leishmaniasis occurrence in response to anthropogenic, climatic and environmental factors at two different scales, in the Neotropical moist forest biome (Amazonian basin and surrounding forest ecosystems) and in the surrounding region of French Guiana. With a consistent data set never used before and a conceptual and methodological framework for interpreting data cases, we obtained risk maps with high statistical support. The predominantly identified human CL risk areas are those where the human impact on the environment is significant, associated with less contributory climatic and ecological factors. For both models this study highlights the importance of considering the anthropogenic drivers for disease risk assessment in human, although CL is mainly linked to the sylvatic and peri-urban cycle in Meso and South America.

## Author summary

Cutaneous leishmaniasis is a vector-borne zoonotic disease with a complex transmission cycle that includes many parasite, vector and host species. This disease continues to pose public health problems worldwide despite the measures put in place. In recent years, methodological tools commonly used in ecology, called ecological niche prediction models, have made it possible to determine the environmental and anthropogenic variables that may be favourable to the presence of the host and vector species communities involved in the cycle and therefore to the presence of certain disease agents. The use of these models, based on the presence of human cases of the disease, can overcome some of the uncertainties concerning the diversity of the vectors and the potential hosts involved in the transmission cycle. This approach of health ecology combining ecology and epidemiology could provide new insights into understanding the cycle of disease transmission and the influence of environmental factors and thus improve the prediction of disease emergence and epidemics. It can be applied to various vector-borne diseases whose transmission cycles are still poorly understood and for which studies classically carried out in epidemiology have not prevented disease progression.

## Introduction

Vector-borne diseases that threaten one-third of the world's population are driven by intertwined socio-economic and environmental factors, such as climate change and modifications of ecosystems through deforestation, conversion of natural habitats to man-made ecosystems and extended urbanisation [1]. To understand these disease agent dynamics, it is necessary to determine (1) the geographic area and associated ecological conditions where the transmission cycle could likely occur, with the infected vectors and host reservoirs, (2) the risk factors that promote transmission to humans and (3) the human communities that are the most exposed to infection hazards on a local scale [1–3]. Landscape ecology may contribute to the knowledge of the influence of biotic and abiotic factors on the presence and dynamics of the vectors and host reservoirs [4]. It also favours the development of spatial models of risk prediction at a relevant geographic scale [5], which finds its theoretical and more practical extensions within the new pathogeography paradigm [6]. These spatial models theoretically make it possible to reveal the geographical areas where the transmission rate of the disease risk is predicted to be the highest by identifying the environmental, climatic and socio-economic risk factors that may expose the most vulnerable individuals and populations to microbial hazards and threats [7,8]. These models may summarise the concept of risk in epidemiology underlying the notions of hazard, exposure and vulnerability. Hazard represents at least the occurrence and distribution of the microbial agent under scrutiny in a geographical area as well as the distribution of vectors, hosts and their interaction. Exposure is related to the probability of an individual or a community being exposed to microbial hazard through recreational or occupational activities. Vulnerability represents the individual and group conditions that make humans more sensitive to infection, e.g., genetic susceptibility or malnourished people [9].

Within the last decade or so, ecological niche models (ENMs) have been proposed in landscape epidemiology to explore the relationships between the potential distribution of vectors or host species reservoirs and environmental variables [10]. The ENMs are used to circumvent gaps in knowledge of species distribution and are based on the occurrence of a species and relevant environmental variables for identifying the most favourable habitats for the establishment and survival of the species of interest [11]. Then they project the relationships over a geographical area to identify non-surveyed areas where there are favourable environmental conditions, and which are propitious for the development and spread of this species. Applied to hosts [12] and vectors [7] of pathogens, it has been possible to better understand the complex influences of spatial heterogeneity and environmental variation on the distributions of species involved in the disease agent transmission cycle, often interpreted as the more likely distribution of the disease agent and hence the disease [13]. Within this framework, the vector-borne disease models show that at larger scales, vectors presence is correlated with climatic and non-climatic factors, with these abiotic factors having a strong influence on vector species range delineation, i.e., the limits of distributional ranges towards more northern areas [7,14]. The influence of anthropic pressures on the environment plays a significant role at more local geographic scales and can unbalance the complex interactions between hosts, vectors and disease agents [15,16]. To properly identify the set of biotic and abiotic conditions suitable for disease maintenance and dispersal, the BAM (biotic, abiotic, movement) framework was proposed [17]. Biotic and abiotic conditions are based on transmission pathways between host and vector communities and shape the geographic and ecologic distributions of the parasite. The movement summarise limitations, accessibility and possible barriers for spreading opportunities. As such, ENMs applied to vector or reservoir-borne infectious diseases may be confounded to the hazards component part in disease risk calculation. This theoretical framework may help to choose the candidate biotic and abiotic variables and the scales at which all these components must be tested to best fit with the biological model. However, relevant movement may be complicated to model.

Today, the development of risk maps for (zoonotic) vector-borne diseases remains difficult for two reasons. First, creating a risk map requires considering the notions of hazard, exposure and vulnerability, in addition to choosing the explanatory variables using the BAM framework. Indeed, the likelihood of contact and contamination between human and host-vectors can vary considerably from one region to another, depending on biodiversity and landscape management programs, education level, health surveillance and control, living conditions, economic resources, etc. [16]. Some anthropogenic variables such as the human footprint (HFP), deforestation, urban expansion and poverty [18] allow studying the vulnerability of human communities. Second, for disease systems with multi-host species and/or multi-vector species [19] it may be unrealistic to model all the actors in systems of such diversified communities of vectors and hosts [20,21]. Identifying explanatory variables and modelling the occurrence of recognised vectors and/or hosts may miss important parts of the infectious disease system, leading to conflicting issues when suitable areas for disease agent establishment are expected to be considered as epidemiologic risks [22–24]. An alternative approach may be to focus on the occurrence of human cases, considering that disease records indicate the circulation of the pathogen, whatever hosts and vectors, including secondary ones, are involved in the disease agent’s life cycle [5,6]. In disease ecology, in the past decade these models relying on human case have shown relevance in identifying more favourable areas for diseases occurrence and risk prediction [25,26].

Thus, species distribution modelling (SDM) with human cases and climatic, environmental and anthropogenic variables may be useful in identifying the different factors influencing the complex disease transmission cycle such as for cutaneous leishmaniasis (CL). CL is caused by a protozoan parasite of the genus *Leishmania* with a complex life cycle involving multiple phlebotomines and mammal species acting as natural vectors and reservoirs, respectively, for the parasite [27,28]. In Meso and Southern Americas, 940,396 new cases of cutaneous (CL) and mucosal leishmaniasis were reported by 17 endemic countries from 2001 to 2017 [29]. American cutaneous leishmaniasis is widespread in the Amazonian Basin and throughout the Neotropical rainforest biome, a region with high biodiversity, and caused by several Leishmaniinae species [30–35]. Within Amazonia, the different *Leishmania* species have a more focal distribution due to their transmission cycles associated with specific ranges of the host reservoirs and vectors [2]. Further, transmission cycles are mainly sylvatic, although urbanisation processes have been reported in some South American countries such as Colombia [34,36]. The sylvatic cycle occurs in forested environments and the rural/domestic cycle occurs mainly in forested-associated human settlements by intra-domiciliary transmission. At the infection focus (a given area where transmission occurred), all components of the cycle must be brought together. Risk models aim to correlate these infection foci with human activities to define the areas that are at high eco-epidemiological risk of infection for humans. However, for leishmaniasis Vélez *et al*. (2017) [2] pointed out that the limit of these infection foci was complex to define due to (1) the high diversity of phlebotomine species and the numerous host species involved in the disease life cycle, (2) the diversity of *Leishmania* species, (3) the complexity of confirming phlebotomine species as vectors and wild mammalians as hosts and (4) the challenge of diagnosing human cases with clinical forms of leishmaniasis. Further, the large geographic extent of the disease and disease agent cycles that may operate in space induce many complex ecological interactions [36] and add uncertainty on the place of infections, which is problematic when models are based on the geolocation of human cases. Last, major anthropogenic disturbances in the Amazonian region impact complex networks of species communities in forest ecosystems; land uses and modifications of the natural habitats are recognised critical factors affecting the mammals and the phlebotomine community's abundance and density [37].

Previous studies have used SDM to map CL occurrence with human cases as input data based on the boosted regression tree (BRT) [14,38] and regression Bayesian modelling [39] showed that climatic parameters acted as the most important predictors of CL distribution at the scale of the South American continent [14,38] and in Brazil only [39]. However, beyond the climatic influence, the level of anthropogenic pressure can act at a finer local scale to influence the disease distribution cycle [40,41].

The aim of the present study was to map the risk of CL based at two different scales in the Amazonian forest and surrounding Neotropical moist forest ecosystems. This geographic area allows working at higher spatial resolution than previously published studies, controlling the influence of bioclimatic factors previously identified as disease occurrence drivers [14,38,39] and likely highlighting a putative role of more local bioecological drivers. We used maximum entropy implemented with the MaxEnt software [42], based on a presence-background ENM, identifying non-linear responses of CL cases to different fine-resolution biotic and abiotic variables at both the Amazonian and French Guianan scales. These two models were run independently and are not assumed to validate each other, but instead are expected to show the extent to which the geographic grain influences the relative importance of contributory variables for the spatial prediction of the disease risk. We used only the official human CL epidemiological records as input data to predict the risk of leishmaniasis occurrence. The cases were geolocated in the health centres, resulting in uncertainty as to the contamination area and geography-biased case reports for this sylvatic disease. To stay within the BAM reasoning framework, we attempted to adapt the model to the real ecological conditions of the CL cycle. To reflect the most likely places of contamination and properly handle the field realities, we randomly distributed the occurrence of cases outside urban centres. By eliminating areas where one is unlikely to find autochthonous CL cases, we succeeded in integrating the movement (M) of the BAM framework. Several redistribution methods made it possible to control the sampling biases related to the uncertainty of case geolocation. The novelty of this work was its redistribution of the occurrences of the disease cases, testing several CL case distribution methodologies, to approach the ecological characteristics of the disease as closely as possible.

## Materials and methods

### Human leishmaniasis cases and study areas

**Fig 1:**
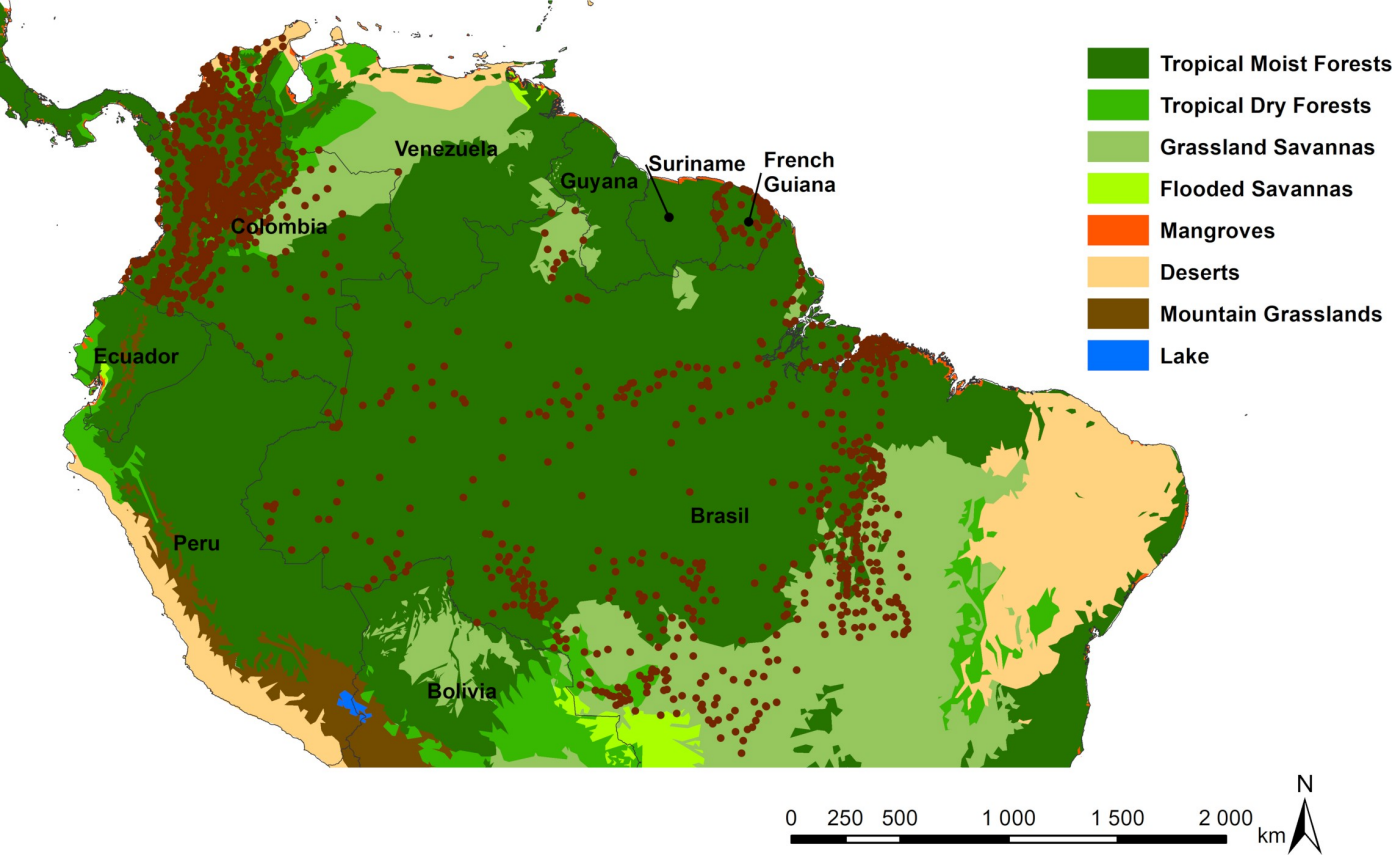
Location of CL actual reported case occurrences illustrated by brown dots, according to different South American northern biomes (Global 200 ecoregions, http://www.worldwildlife.org [44]), and the administrative borders. Final map produced with ArcGis 10.4.

For the Amazonian model, we used a total of 149,368 human CL cases referenced in 1415 localities from Brazil, Colombia and French Guiana. These case records were predominantly located in the same large Neotropical moist forest biome that encompasses the Amazonian basin, the Guiana shield, and north-west forests of South America (Fig 1). For Brazil, 75,441 CL cases, reported from 2007 to 2015, spread across 444 localities in the Amazonian states of Acre, Rondônia, Tocantins, Pará, Roraima, Amapá, Mato Grosso and Amazonas were obtained from the Secretaria de Vigilância em Saúde-SVS (Secretary of Surveillance in Health) from the Brazilian Ministry of Health. The data were validated by the Technical Group of Leishmaniasis, the Coordenação Geral de Doenças Transmissíveis (CGDT), the Departamento de Vigilância de Doenças Transmissíveis (DEVIT) and by the Secretaria de Vigilância em Saúde (SVS) of the Ministério da Saúde. Input data for CL for these states were the place of infection at the municipality scale. In Colombia, 73,479 cases were spread across 882 localities in all the 32 departments of Colombia from 2007 to 2015. Colombian data were extracted from the SIVIGILA (National Public Health Surveillance System) website, which gathers cases of the various diseases that require mandatory reporting. CL data were validated by the Grupo de Investigaciones Microbiológicas-UR (GIMUR) from Universidad del Rosario, as reported elsewhere [43]. In French Guiana the 448 cases distributed in 89 localities come from patients in consultation for suspected leishmaniasis at the LHUPM (Laboratoire Hospitalo-Universitaire de Parasitologie et Mycologie) and in the country’s different health centres, between 2008 and 2015.

We chose not to include cases from Venezuela, Suriname, Guyana, Bolivia and Peru, because we had no access to official cases coming from health centres that could be considered as non-biased public data.

### Ethics statement

We report a geospatial analysis of CL data. For Colombia and Brazil, the data were readily obtained from existing public access databases (Colombia: SIVIGILA, and Brazil: SINAN). For French Guiana, we report the cases from the database already published in Simon *et al*. (2017) [45]. For all data, the information that identifies the patient was anonymised in the databases and there is no need for ethical considerations.

### Parameters in the models

All data were processed in ArcGis 10.4 [46]. All variables were used at the resolution of 30 arc-seconds (~1 km^2^) for the Amazonian and French Guiana models. Geographic variables available at another resolution and vectorized variables were resampled at 30 arc-seconds using the nearest neighbour joining method, implemented with ArcGis 10.4. The bioclimatic, environmental and anthropogenic variables are given in Table 1, with their initial resolution.

**Table 1:** Bioclimatic, environmental and anthropogenic variables used for the different geographical models.

Model	Category	Variables	Initial resolution
Amazonian—French Guiana	Bioclimatic—Abiotic	(bio1) Annual Mean Temperature	30 arc-sec (~1 km)
Amazonian—French Guiana	Bioclimatic—Abiotic	(bio2) Mean Diurnal Range (Mean of monthly (max temp—min temp))	30 arc-sec (~1 km)
Amazonian—French Guiana	Bioclimatic—Abiotic	(bio3) Isothermality (bio2/bio7) (* 100)	30 arc-sec (~1 km)
Amazonian—French Guiana	Bioclimatic—Abiotic	(bio4) Temperature Seasonality (standard deviation *100)	30 arc-sec (~1 km)
Amazonian—French Guiana	Bioclimatic—Abiotic	(bio5) Max Temperature of the Warmest Month	30 arc-sec (~1 km)
Amazonian—French Guiana	Bioclimatic—Abiotic	(bio6) Min Temperature of the Coldest Month	30 arc-sec (~1 km)
Amazonian—French Guiana	Bioclimatic—Abiotic	(bio7) Annual Temperature Range (bio5-bio6)	30 arc-sec (~1 km)
Amazonian—French Guiana	Bioclimatic—Abiotic	(bio8) Mean Temperature of the Wettest Quarter	30 arc-sec (~1 km)
Amazonian—French Guiana	Bioclimatic—Abiotic	(bio9) Mean Temperature of the Driest Quarter	30 arc-sec (~1 km)
Amazonian—French Guiana	Bioclimatic—Abiotic	(bio10) Mean Temperature of the Warmest Quarter	30 arc-sec (~1 km)
Amazonian—French Guiana	Bioclimatic—Abiotic	(bio11) Mean Temperature of the Coldest Quarter	30 arc-sec (~1 km)
Amazonian—French Guiana	Bioclimatic—Abiotic	(bio12) Annual Precipitation	30 arc-sec (~1 km)
Amazonian—French Guiana	Bioclimatic—Abiotic	(bio13) Precipitation of the Wettest Month	30 arc-sec (~1 km)
Amazonian—French Guiana	Bioclimatic—Abiotic	(bio14) Precipitation of the Driest Month	30 arc-sec (~1 km)
Amazonian—French Guiana	Bioclimatic—Abiotic	(bio15) Precipitation Seasonality (Coefficient of Variation)	30 arc-sec (~1 km)
Amazonian—French Guiana	Bioclimatic—Abiotic	(bio16) Precipitation of the Wettest Quarter	30 arc-sec (~1 km)
Amazonian—French Guiana	Bioclimatic—Abiotic	(bio17) Precipitation of the Driest Quarter	30 arc-sec (~1 km)
Amazonian—French Guiana	Bioclimatic—Abiotic	(bio18) Precipitation of the Warmest Quarter	30 arc-sec (~1 km)
Amazonian—French Guiana	Bioclimatic—Abiotic	(bio19) Precipitation of the Coldest Quarter	30 arc-sec (~1 km)
French Guiana	Bioclimatic—Abiotic	Cloud coverage	1 km
Amazonian—French Guiana	Environmental—Abiotic	Elevation (SRTM)	30 arc-sec (~1 km)
French Guiana	Environmental—Abiotic	Distance to a relief at least of 500 meters	vector
French Guiana	Environmental—Abiotic	Distance to forest edge	vector
French Guiana	Environmental—Abiotic	Distance to river courses	vector
Amazonian—French Guiana	Environmental—Biotic	Aboveground biomass	1 km
Amazonian—French Guiana	Environmental—Biotic	Forest canopy height	0,6 mile (1 km)
French Guiana	Environmental—Biotic	Percentage of the cell covered by high forest	vector
Amazonian	Environmental—Biotic	Richness in mammal species	30 arc-sec (~1 km)
Amazonian	Anthropic—Biotic	Population density	30 sec (~1 km)
Amazonian	Anthropic—Biotic	Poverty	1 km
Amazonian—French Guiana	Anthropic—Biotic	(HFP) Human footprint	30 sec (~1 km)
French Guiana	Anthropic—Abiotic	Density of tracks and road network	vector

In total, 26 variables were used for the Amazonian model including 19 bioclimatic variables from WorldClim2, three anthropogenic variables with the population density, the human poverty and the human footprint (HFP) and four environmental variables: the biomass aboveground, elevation, forest canopy height and species richness in mammals. For French Guiana, we used the same 19 bioclimatic variables that for the Amazonian model, plus a cloud cover variable. The same environmental variables were used as for the Amazon model with in addition, the percentage of the cell covered by high forest, the distance to river courses, the distance to forest edge and the distance to a relief at least of 500 meters. However, we did not have the species richness variable in mammals for this last model. Two anthropogenic variables were used, the density of tracks and road network and HFP; we used a specific HFP developed for French Guiana, which has a higher level of detail and a more recent update than for the Amazonian HFP variable. The detailed information and sources of the variables used for both models is available in supplementary method (S1 Method).

### Model implementation and processing

#### Using MaxEnt

The aim was to identify the response curves of the likelihood of case occurrence in relation to the most contributing explanatory variables and then to highlight areas with the most favourable predictors for disease occurrence and risk. To examine how our range of environmental and anthropogenic factors may influence leishmaniasis occurrence, we used a generalised additive model (GAM) to model the occurrence of human CL cases as input data, under the software MaxEnt, version 3.3.3k [42]. The main benefit of GAMs over techniques such as logistic regression and other generalised linear models is that they use regression splines to estimate the response curves of different predictor variables and can thus learn their non-linear contributions to the dependent variable. Models were fitted with all possible variables of interest. MaxEnt is a highly confident presence-background model based on maximum entropy that does not require real absence data [42]. It belongs to machine learning modelling, which builds a two-step model: the first step is based on a part of the input occurrence data and identifies the best explanatory function for the occurrence of disease cases according to each environmental variable, and then sums this up. These functions create a first learning model. Second, a general model is created with all these functions, using all cases of occurrence. MaxEnt does not require true absence data because it generates background data that we set at 10,000 background points for this study. For the replicated run type, we used the subsampling strategy with 30% of cases used for training, with 1,000,000 iterations. To consolidate the final model, we made ten replications, without using threshold values and with response curves and jackknife analyses as output data. MaxEnt generates a map where the likelihood of favourable areas for leishmaniasis transmission to humans ranges from 0.0 to 1.0. The post-processing of the map was done in ArcMap 10.4.

#### Variable selection

We used a backward stepwise selection procedure to exclude the explanatory variables that did not contribute to improving the model. The individual components were evaluated under the jackknife test and we stopped removing variables when all variables had a percent contribution greater than 5%. The quality model was evaluated with the area under the curve (AUC) and the omission rate [47]. Thus, models with the lowest training and test omission rates and with the highest AUC were retained as the best minimal models. Before validating the final minimal model, we checked that the remaining variables were not spatially autocorrelated by performing a Pearson test implemented with SDM toolbox 2.0 [48].

#### Model evaluation

The reliability of the final minimal model was evaluated using a null model [49], which is based on the comparison between the AUC of the model created with the CL cases reported and the AUC of the models with the same number of randomly distributed cases of CL. We therefore generated 100 models with randomly distributed points. Resulting AUCs were classified in ascending order, and we compared the AUC of the final leishmaniasis model. If the AUC of the final leishmaniasis model was greater than the 95th value, the final model was considered better than a random model with a confidence interval greater than 95%. To study the potential sampling bias of the models, we tested two additional models, excluding cases. For the first one we arbitrarily removed the Colombian cases and for the second one we removed the French Guianan cases. The results are available in Supplemental results.

### Data preparation for modelling

#### Weighting cases

Since the geographically close occurrences are likely to be spatially autocorrelated, MaxEnt requires rarefaction of input data when data sets are large, minimizing the number of cases per cell (i.e. 1 km^2^ in our study) before the model is implemented. The resampling strategy thus allowed mitigating the model’s over-learning in oversampled areas. For the Amazon, we averaged the number of cases in each locality recorded yearly. Then we arbitrarily created three classes of weighted occurrences: localities with one to ten cases (*n* = 1,115) were represented by one point, localities with 11–100 cases were weighted with two points (*n* = 266) and localities with more than 101 cases (*n* = 102) with four points. A total of 149,368 cases for the Amazonian model were weighted, giving a final 4,280 CL cases to use for model development. For the French Guiana model, we did not create classes but only kept the average number of cases per year, ranging from one to five, for each locality. Finally, 111 CL cases were used to elaborate the final model.

#### Point distribution methodology

In official reports, geolocation of CL cases was most often provided at the level of urban centres, administratively referenced, which is likely not the exact place of infection where people were infected. CL is a mainly sylvatic disease and we wished to randomly redistribute these cases in the most likely contamination areas. This redistribution allowed us to integrate the movement (M) of the BAM diagram into the model, excluding areas where finding the pathogen is very unlikely. Weighted case occurrences were distributed with ArcGis 10.4. We first defined an exclusion zone in the most urbanised areas based on the score of the HFP layer and we created a distribution zone, which represents the likely contamination zone to distribute the points of presence, around the exclusion zone. The exclusion values of HFP were selected based on satellite imagery of large urban centres. We adapted the scales of the exclusion and distribution zones according to the two geographic models. To control for resampling bias of this resampling we developed three different distribution methods (Table 2, with detailed procedure provided in Supplemental method). These three methods allowed us to (i) test whether the exclusion zones of models 1 and 2 excluded the urban centres with a high HFP, (ii) determine whether it was important to adapt the point distribution area proportionally between the two French Guiana-Amazonian scales for model 3 and (iii) test whether or not the distribution of classes made with the HFP in model 1 biased the random distribution of points. Last, to avoid pseudo-replication problems in modelling and over-representation of independent variables, the points of CL occurrence were all spaced a minimum 1 km^2^ apart.

**Table 2:** Creation of HFP classes and methods of distribution of the occurrence points in the exclusion and distribution buffers for the Amazonian and French Guiana models. The size indicates the radii of buffers in kilometres.

	Amazonia		French Guiana		
	Exclusion buffer (km)	Distribution buffer (km)	Exclusion buffer (km)	Distribution buffer (km)	HFP
**Method 1**	0.5	3	0.5	3	0–29
	2.5	5	1	5	30–50
	7.5	10	2	6	51–90
**Method 2**	HFP > 50	3	HFP > 40	3	
	HFP > 50	5	HFP > 40	5	
	HFP > 50	10	HFP > 40	6	
**Method 3**	HFP > 50 + 0.5	3	HFP > 40 + 0.5	3	
	HFP > 50 + 2.5	12.5	HFP > 40 + 1	5	
	HFP > 50 + 7.5	22.5	HFP > 40+ 2	6	

## Results

The three different methods for Amazonian and French Guiana models show only very few differences in their respective AUCs, and the occurrence of CL cases is explained by the same set of environmental and anthropogenic variables (S1 Table). Test omission rates are null at the minimum training presence threshold for training datasets (rate = 0.000 for all methods 1, 2 and 3) and very low for the test omission (rate method 1 = 0.0005, rate method 2 = 0.0017, rate method 3 = 0.0008). For the 10th percentile training presence threshold the training rate = 0.099 and the test rate = 0.11 for the three methods. Pair-wise comparisons, using non-parametric tests, show no deviation from the null hypothesis of differences across the three Amazonian ENM methods (for latitude, *Z* [0.688; 0.697; 0.941], *df* [1241; 1267; 1336] and *p* [0.491; 0.486; 0.347] for comparison between method1-method2, method1-method3, and method2-method3, respectively; for longitude, *Z* [1.122, 0.795, −0.468], *df* [1244; 1255; 1343] and *p* [0.262; 0.427; 0.640] for the same comparisons). Consequently, our three disease case distribution methods do not influence the quality of the models. For the analysis of the results, hereafter we only consider the models with the distribution method leading to the best AUC for French Guiana and Amazonia.

### Amazonian model

Method 2 of the distribution of the points led to the best AUC score (0.842; 95^th^ ranked AUC value for null model = 0.5073) (S1 Table). The five variables explaining the probability of occurrence of CL cases best were, human population density (30.8% of the contribution), HFP (30.2%), Bioclim 4 (seasonal temperature; 18.9%), mammalian species richness (13.8%) and aboveground biomass (6.3%). For the jackknife test the variable with the highest gain when used alone was population density, which therefore appears to contain the most useful information by itself (S1 Fig). The variable that most decreases the gain when it is omitted is Bioclim 4, which therefore appears to have the most information that is not present in the other four variables (S1 Fig).

**Fig 2:**
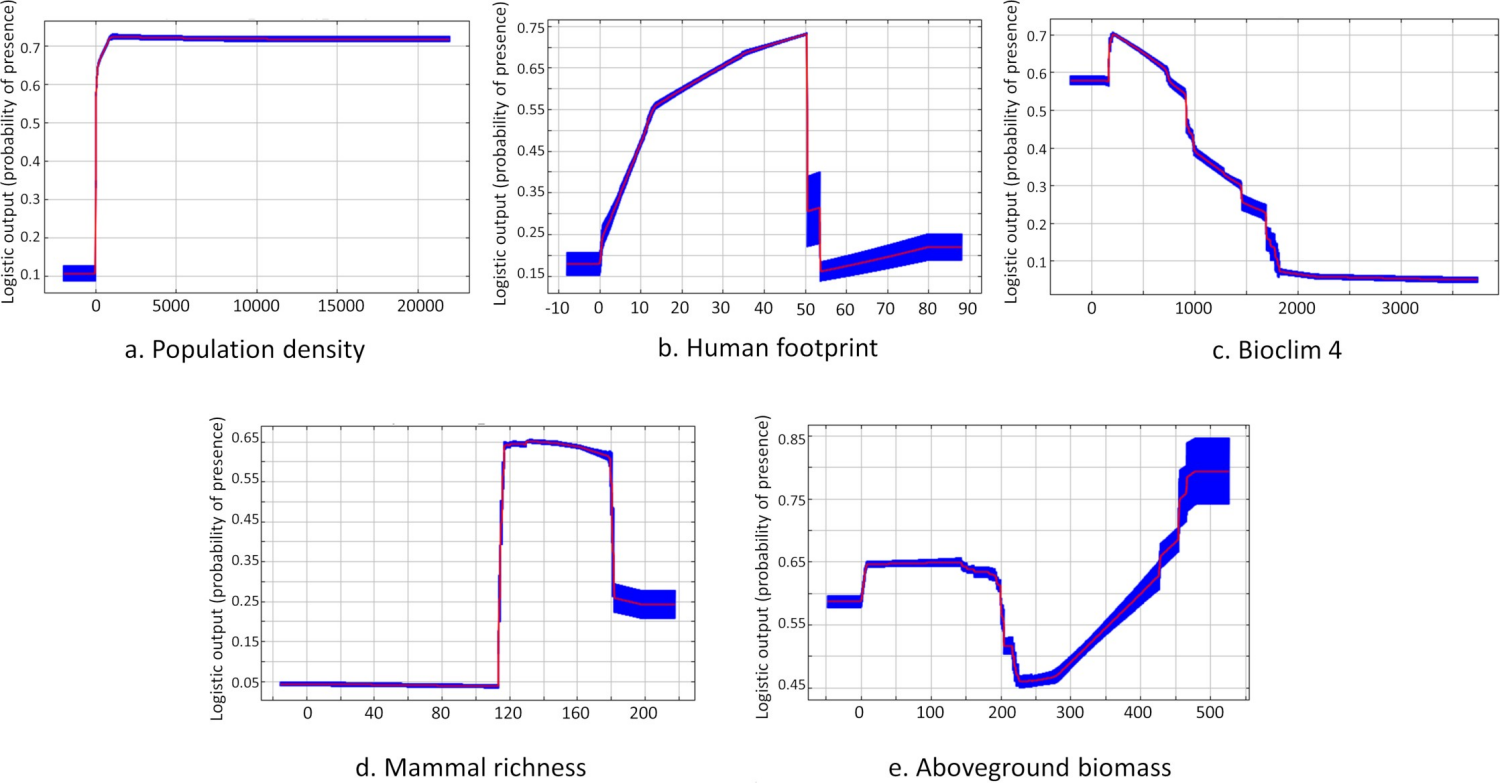
Response curve of cutaneous leishmaniasis probability of occurrence for the four most contributing variables in Amazonia with population density (a), human footprint (b), Bioclim 4 (c), mammal richness (d) and aboveground biomass (e).

The likelihood of occurrences does not vary whatever the population density (Fig 2A). The likelihood of occurrence increases sharply to a HFP value of about 50, then decreases sharply (Fig 2B). This decrease can be attributed to our method of distributing case occurrences for high HFP values, excluding the more anthropised areas and large urban centres in the Amazon (values above 51) where transmission of CL is unlikely to occur given the ecology of the CL transmission cycles. The likelihood of case occurrence decreases rapidly as the seasonal temperature variation (Bioclim 4) increases (Fig 2C). The likelihood of occurrence of cases with mammal species richness looks like a bell-shaped curve: it abruptly increases near 110 species, since low-richness areas indicate either non-forested habitats, where CL does not occur, or disturbed forest habitats; the occurrence then decreases for the highest mammal richness values, those associated with very remote, species-rich and restricted Amazonian regions where, at least, no CL human cases are reported (Fig 2D). Concerning the aboveground biomass, the likelihood of case occurrence is stable, then decreases over a very small interval of the variable, between 200 and 250 tons/ha, and finally increases when the values of the variable increase.

**Fig 3:**
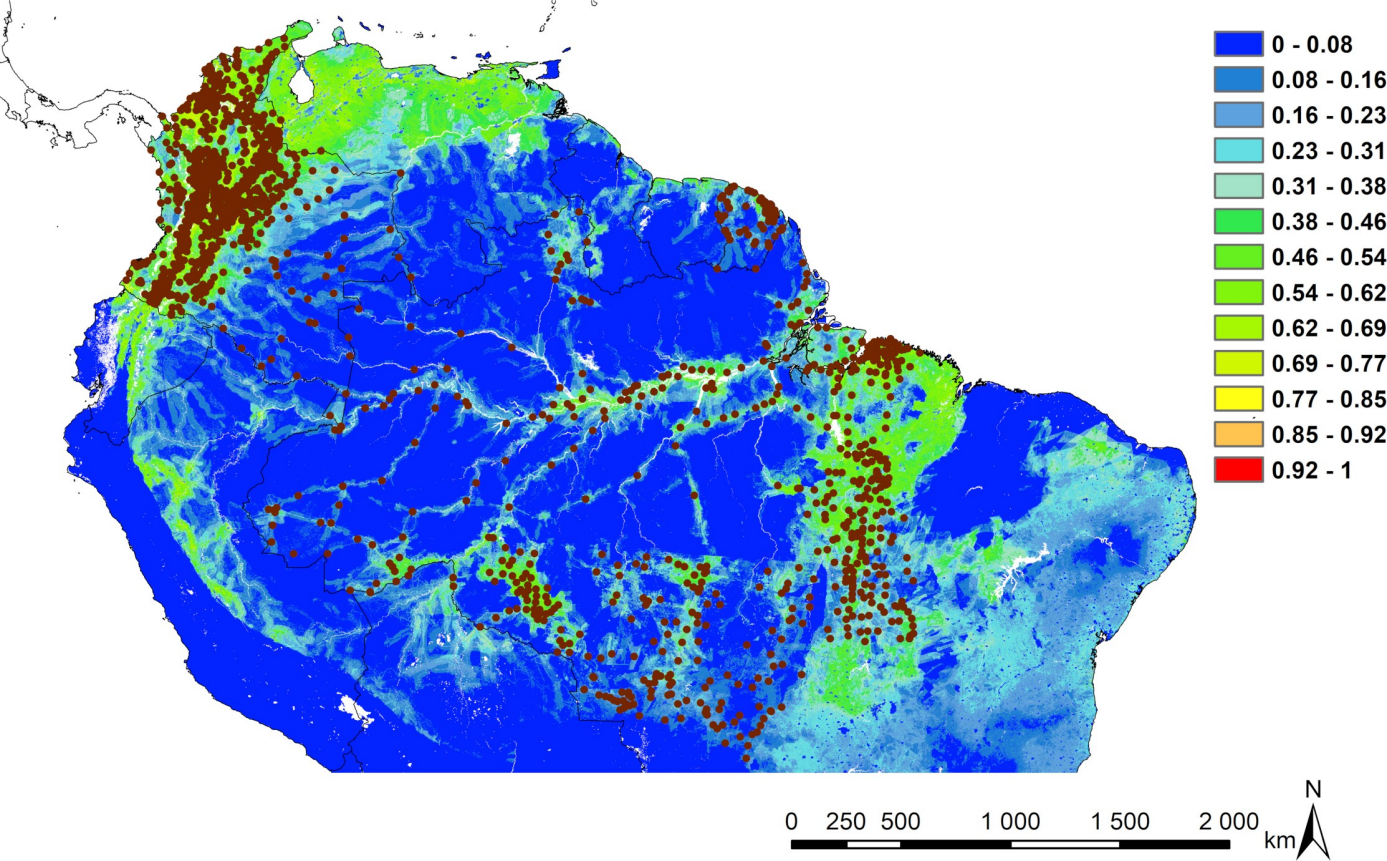
Risk map for the Amazonian model. The risk area prediction maps are calculated using the Habitat Suitability Index (HSI) calculated between 0 and 1. Increasing suitability follows a gradient from colder to warmer colours. Cases of CL are represented by brown dots. Map made with ArcMap 10.4.

The predicted risk map is driven mainly by population density and HFP, showing disturbed forest areas and large nuclei of human populations as foci potentially at risk for leishmaniasis transmission to human populations living in these contexts. The north-northwest of South America, mainly Venezuela, and the south-eastern part of the Amazon basin, notably near the south of the delta area, appear as the most at-risk areas for leishmaniasis transmission according to the explanatory variables retained in the models (Fig 3).

### French Guianan model

The model with distribution method 2 had the best AUC (0.885, null model = 0.5491) (S1 Table). The best AUC score was obtained with four explanatory variables that included two climatic variables (Bioclim 2 and 16; mean diurnal range of temperature and the precipitation of the wettest quarter, respectively), one anthropogenic variable (HFP) and one environmental variable (distance to forest relief), with overall the most significant contribution being HFP with 70.1% of the total explanation. The jackknife test training shows that the explanatory variable with greatest gain when used alone and that decreases the gain the most when omitted is HFP. Jackknife analysis was performed to test the importance of each of the variables retained. Bioclim variables 2 and 16 contributed 9.2% and 15.4% of the total explanation, respectively. The last variable distance to a relief of at least 500 m seemed to contribute very little to the model (5.3%), but the jackknife test showed a decrease in AUC when the variable was not present in the training and the test (S2 Fig).

**Fig 4:**
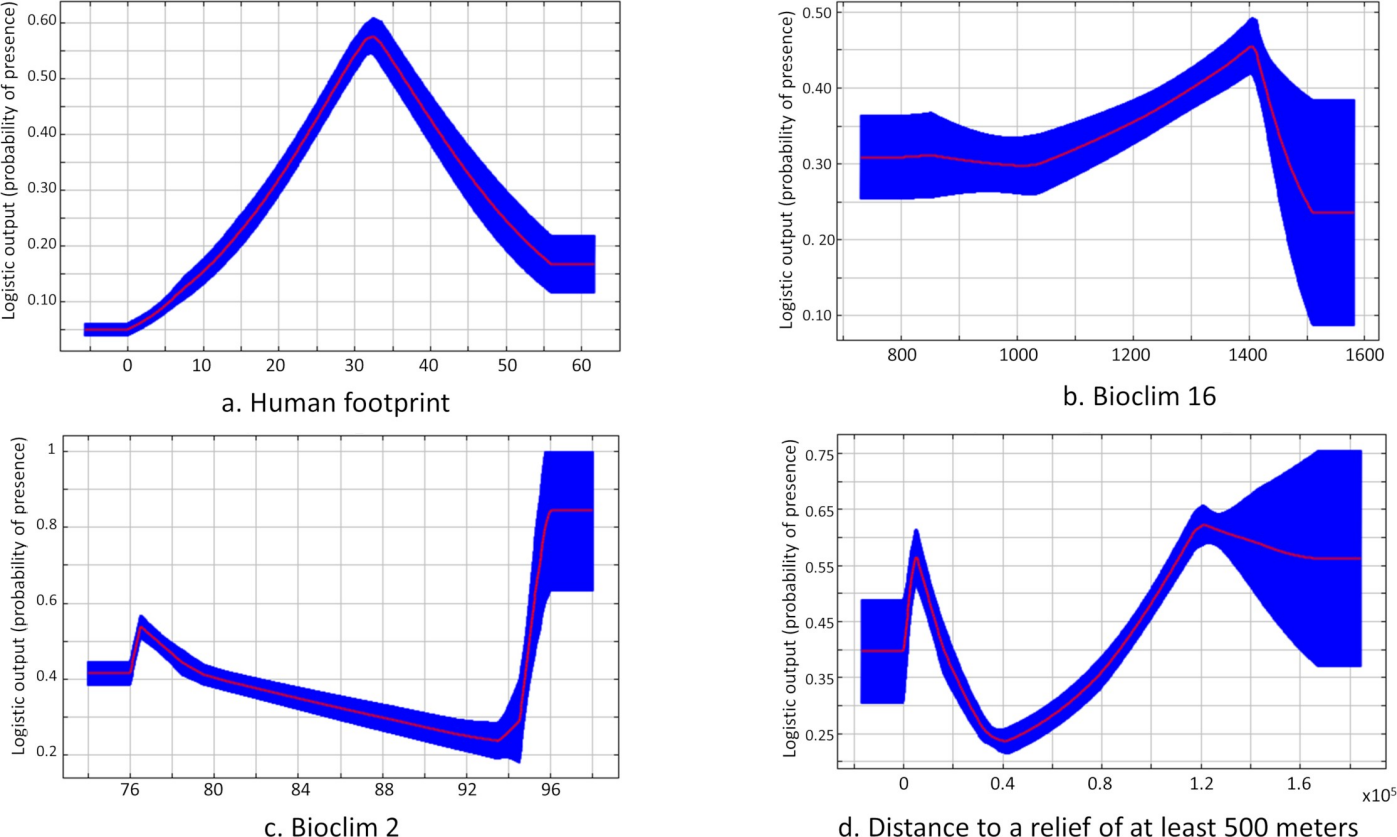
Response curve of disease occurrence for the four variables contributing most in French Guiana with HFP (a), Bioclim 16 (b), Bioclim 2 (c) and distance to relief of at least 500 m (d).

The likelihood of occurrence increases with HFP until 35–40 and then it decreases according to a bell-shaped curve. This decrease is directly related to the point distribution of method 2 since areas with HFP > 40 were excluded from contamination areas (Fig 4A). For Bioclim 16, the likelihood of occurrence slightly increases with precipitation of the wettest quarter, indicating that the occurrence of cases increases monotonically during the rainy season in this region (Fig 4B) and then drops for the highest values of precipitation of the wettest quarter. The response of the mean diurnal range variable (Bioclim 2) shows that the likelihood of occurrence slightly decreases as the temperature amplitude increases and then sharply rises to reach a plateau for the highest values of Bioclim 2 (Fig 4C). When the amplitude is the highest, there is a sharp increase in the likelihood of cases occurring, as explained by several cases of CL in the eastern part of the French Guiana region. The response curve of the distance to relief of at least 500 m variable shows that occurrence is high at 500 m and then drops off rapidly and increases gradually at lower altitudes (Fig 4D).

**Fig 5:**
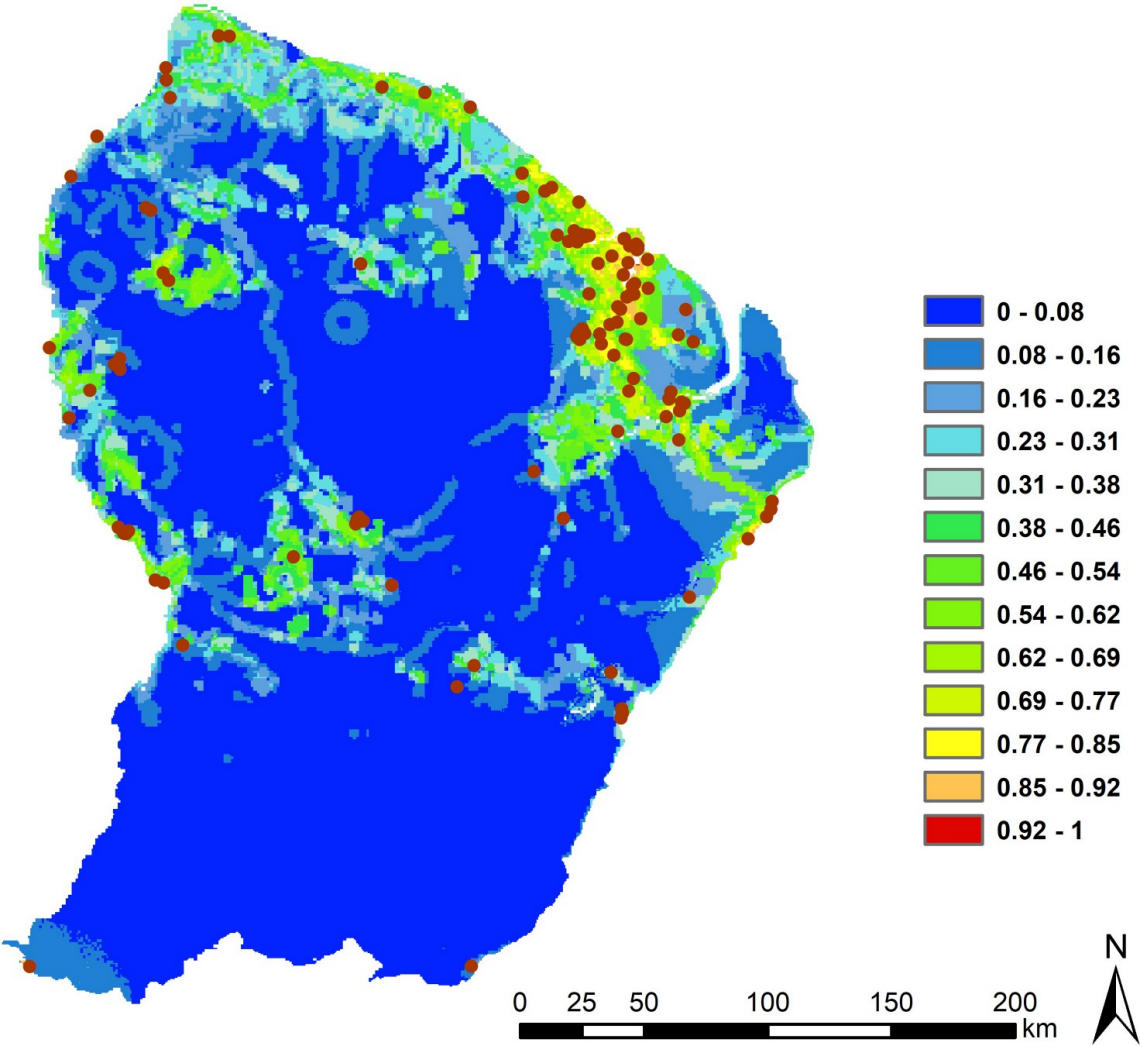
Risk map for the French Guiana model. Increasing suitability follows a gradient from colder to warmer colours. Cases of CL are represented in brown. Map made with ArcMap 10.4.

The risk map shows that prediction for CL transmission is higher where the HFP index is high, i.e. anthropogenic activities (hunting, logging, development of activities and housing at edges) are most common (Fig 5).

## Discussion

The potential input value of ecological niche modelling (ENM) for spatial epidemiology is widely accepted for generating risk maps and answering ecological and distributional questions related to the disease system, its persistence (i.e. endemicity and high spots) and spread (i.e. development of epidemics) [50]. However, to design a good study it is necessary to consider a set of issues related to the type and use of occurrence data, the relevance of explanatory variables and the spatial extent of the model, and to ensure adequate statistical support [50]. The aim of the present study was to propose a risk map of cutaneous leishmaniasis (CL) based on anthropogenic, climatic and environmental factors at two different scales, in the Neotropical moist forest biome (Amazonian basin and surrounding forest ecosystems) and in French Guiana. The models were created using a presence-background ENM constructed on human disease cases in the biotic, abiotic, movement (BAM) framework [17]. Compared to previous studies, one novelty in the present investigation was the redistribution of points to try consider the known ecological characters of the CL cycle and to integrate the movement component of BAM [51–53], complementary to the biotic and abiotic components more widely used The reduction and the redistribution of cases limited the over-representation of certain environmental conditions and allowed us to focus on the more favourable zones of infection, a key issue when dealing with georeferenced data extracted from official reports [54]. Based on a consistent data set never used before and a conceptual and methodological framework for interpreting a quantitative analysis of cases, we obtained risk maps with high statistical supports. This approach thus increases the potential of ENM for further investigation in modern pathogeography [6].

### Environmental variables

At the beginning of this study, the set of initial variables tested was large enough to encompass all the ecological complexity of the CL life cycle.

In agreement with previous studies using human CL occurrence data [14,38,39], the variable contributing most to the Amazonian model were two anthropogenic variables, i.e. population density and HFP, followed by seasonal temperature, mammal species richness and aboveground biomass. At French Guiana scale, the variables explaining the greatest number of cases were HFP, followed by precipitation in the wettest quarter (Bioclim 2) and the mean diurnal range of temperature (Bioclim 16).

At the Amazonian large scale, the presence of four biotic variables with wild mammal species richness, population density, HFP and aboveground biomass show the likelihood of increased case occurrence when all these parameters also increase. Several studies have shown that changes in human activities with landscape management in rural areas may affect the population dynamics and distribution of phlebotomine species in Amazonia [41,55,56]. The response of the seasonal temperature indicates that CL cases are more likely to occur in geographical areas with the least amplitude in seasonal variation. This is not surprising and lends support to the absence of CL cases in the Andes Mountains, with their present unfavourable meteorological and ecological conditions for phlebotomine vectors [33]. Although this observation is ecologically consistent for a large-scale study, it does not add information on climatic factors favouring the risk in the Amazonia biome. Here, unlike the results of previous studies [14,38], the contribution of rainfall remained below 5%, probably because the model is run in the same biome where precipitation has no significant impact on the risk of CL transmission. Wild mammal species richness and aboveground biomass are reminders that the involvement of mammalian hosts and the ecology of the vector are also important biotics drivers to be considered in assessing the risk of CL [57]. Interestingly, in French Guiana, the likelihood of case occurrence is also mainly driven by the biotic HFP variable, with cases increasing as HFP rises. Although environmental policies in this region are very protective [58], pressures on forest ecosystems have changed over the last few decades. Today, 86.2% of CL cases reported are due to *L*. *guyanensis* whose the life cycle is mainly sylvatic, but an increase in cases due to *L*. *braziliensis* has been observed in recent years [45]. The ecology of *L*. *braziliensis* has been assimilated with disturbed and peri-domestic forest habitats in several parts of Amazonia [37]. For this model, the HFP biotic variable probably provided a better account for anthropogenic modification on the environment given its finer resolution and more up-to-date data than those used for the entire Amazonian region [59].

For French Guiana, we observed a probability of an increase in CL case occurrence when the precipitation of the wettest quarter and mean diurnal range increased, confirming the importance of these climate variables in the Amazon basin regardless of the scale chosen. Indeed, in French Guiana a large majority of cases are in the north-east region where precipitation and mean diurnal temperature variations are the greatest. This increase can potentially be explained by the climatic conditions, which are more favourable for vector proliferation, and by the more extensive anthropogenic activities related to the forest [59]. For the response curve of the variable representing the distance to a relief of more than 500 m, the probability of cases occurring is higher on the 500-m reliefs and when one moves away from these reliefs. This result may reflect the high biological diversity of phlebotomine species with different altitudinal distributions as we observed in many regions of Southern America. Ready *et al*. [60] showed the presence of *Psychodopygus wellcomei*, the main vector of *L*. *(V*.*) braziliensis* in Amazonia, at altitudes over 500 m and then the sharp drop in the probability of occurrence of CL cases and its consistent increase can reflect the ecological requirement of vectors in French Guiana.

### Risk map

The risk map obtained for the Amazonian model is relatively similar to the at-risk areas highlighted by a previous study at the South American scale [14]. However, it differs from the map obtained by Purse *et al*. [38] where the entire Amazon basin was found at risk. In the present study, the AUC, omission test and the null model suggest that the predictions are reliable. The predominantly identified risk areas are described where the human impact on the environment is substantial, i.e., close to urban centres and along roads and rivers where human populations are concentrated.

Venezuela, north-east of Brazil, and northern Bolivia emerge as potential at-risk areas while no case of CL in this region was used in the model. The data currently available on CL indicate that cases have been identified in these areas [61], although they are not being included as input data, suggesting that the model did not make a significant commission error. In Colombia, the states in the south-east did not come out as a potentially high-risk area. This result seems to contradict the recent study conducted by Herrera *et al*. [43], which indicated that these states had the highest incidence and number of cases in the country. This failure may be explained by the limit of spatial ENM when working with quantitative data. Despite a very high number of reported CL cases in this region, the number of cities and the population density remain very low. However, ENMs handle quantitative data such as prevalence, because the information is retained at the pixel scale and whatever the number of cases in one pixel, it is saturated with the first reported case. Despite our procedure to create a buffer zone to randomly disperse cases, cases and substantially increase the number of available pixels to distribute the cases, the model still gives greater importance to areas where the spatial occurrence of cases is widely distributed.

For French Guiana, this is the first study to propose a high-resolution risk map based on precisely geolocalised cases. For this European territory, high-risk areas are located where the anthropogenic pressures on habitats are the strongest. A risk zone appears on the map in the west of this region despite the absence of cases, suggesting under-reported and/or under-diagnosed cases. French Guiana is a region where deforestation, hunting, forestry activities, and legal and illegal gold panning have increased in recent years [62]. This information, collected on the importance of the influence of human activities in increasing the risk of this disease, as well as the numerous studies carried out on the possible anthropisation of the vector cycle as shown in Colombia [34] and Manaus, Brazil [63], suggest that human activities in the rainforest in the Amazon and French Guiana could promote a peri-domestication of the CL disease cycle. Also, throughout Amazonia, people could be infected in peri-urban forest fragments with great canopy cover, which is essential for maintenance of the *Leishmania* vector/reservoir species diversity and abundance [64–66].

### Limits

The methodology of this study is based on satellite imagery and correlative analyses, but it remains a visual assessment. It also excludes that the cycles could occur in anthropised and highly disturbed habitats. Indeed, in Colombia CL is linked to the urban cycle [34] and in the largest Amazonian cities such as Belém, CL is associated with small forest fragments surrounded by an urban area and where (phlebotomine) putative vectors may sustain [64]. Consequently, it may be interesting to retain relatively high values of HFP in order not to completely obscure the likelihood of local peri-domestication of CL. Another limitation of our study is that some areas of the Amazon biome are not considered at risk while we do know the existence of CL cases, as in Peru and Bolivia. Heterogeneity in the availability of our data increases the models’ omission rate, but we favoured data that were reliable and retrieved directly from the public health database for each country. Unfortunately, it was possible to find this kind of data for only two countries, i.e., Colombia and Brazil, and for the French Guiana region. We also attempted to obtain the most updated variables for the Amazonian model, but some are not updated over the period when the cases occurred, so the environmental data are not necessarily concomitant with the case occurrence period. In addition, we are aware that the models are highly dependent on the input variables and spatial scaling, so risk maps produced with large-scale data and models should not be extrapolated for more restricted geographical areas; risk maps are first context- and space-dependent.

### Conclusion

Modelling a parasite system that is based on several species of hosts and reservoirs requires considering relevant biotic and abiotic variables summarising the ecological conditions in which the transmission cycle takes place. For this complex issue, the BAM diagram may help to select the variables and the scale of study. Finally, for both models (Amazonia and French Guiana) this study highlighted the importance of considering the anthropogenic drivers for risk assessment. This conclusion differs from that proposed by Pigott and collaborators, [14] who argued that climatic conditions were the main driver of CL case distribution in South America. The adequate choice of the spatial scale under scrutiny, in accordance with the variables explored, can be a major determinant in the discrepancy that we observed between Pigott *et al*. and our present results. Therefore, risk mapping should not be made without considering variables representing the vulnerability of human individuals and communities to the disease and further add to the importance of an appropriate scaling when designing ENM studies [50,67]. Generally, coarse-scale studies appear to favour the importance of climatic variables in explaining infectious disease presence and spatial distribution [68]. This pattern has already been referred to as Eltonian Noise Hypothesis [69] which assumes that local biological interactions or microhabitat biotic conditions required by a specific parasite cycle should not affect niche estimates at coarse scales [19].

### Perspectives

Many studies have attempted to make future projections of climate change on vector-borne diseases to determine the factors favouring disease emergence and to predict the dispersal of infectious disease agents. For diseases whose transmission cycles are confined to restricted geographic areas, it is likely that the small-scale human impact firstly may influence spatial expansion or regression of these diseases. With the methodological framework proposed here and with fine-scale and updated variables on anthropogenic disturbances, ENMs remain a valuable tool to determine local factors that are the drivers of parasite transmission and may help relevant decision-making by health authorities. Every ENM study that uses risk modelling should target the proper scale based on these elements. This statement can be extended most particularly to the *Leishmania* ecological system. In French Guiana, the CL system is mainly represented by *L*. *guyanensis* and *Nyssomyia umbratilis* with Xenarthran species acting as major host reservoirs [31,45], while this cannot be identical for other pan-Amazon regions with other species involved in the cycle [34,70]. The relevance of developing future models of CL risks with only climatic variables is questionable. Indeed, it is likely that the policy and economic decisions with their cascading impacts on poverty, hygiene, war, displacement of populations, etc., and short-term local planning strategies [71] will have a more direct and immediate impact on biodiversity and their interactions with disease components. This is particularly true in regions where the expected climatic variations will remain low compared to the impact of microclimates created, for example, by the creation of hydroelectric dams [40], the burden of extensive agriculture [72] or the effects of edge habitats [73]. These anthropogenic factors will remain extremely difficult to control in the future and will continue to challenge the relevance of predictive models, whatever the ongoing methodological improvements and the quality of the data used as independent variables in models.

## Supporting information

S1 Method Detailed information on the variables used for the Amazonian and French Guianan models. (DOCX)

S1 Results Results of risk models to evaluate sampling biases without occurrence data from Colombian and French Guiana cases. (DOCX)

S1 Table AUC results for the three models for Amazonia and French Guiana and the null models as well as explanatory environmental and human variables. The explanatory variables are ranked in order of importance of their contribution to the model.
(XLSX)

S1 Fig Jackknife test results for the Amazonian model with CL case distribution method 2. These tests represent the contribution of each variable independently of the others. Values shown are averages over replicate runs.
(TIF)

S2 Fig Jackknife test result for the French Guiana model with CL case distribution method 2. These tests represent the contribution of each variable independently of the others.
(TIF)
