# Supplementary material for: Ecological niche modelling for predicting the risk of cutaneous leishmaniasis in the Neotropical moist forest biome
Source: PLoS Negl Trop Dis. 2019 Aug 14;13(8):e0007629. doi: 10.1371/journal.pntd.0007629 (PMC6693739; doi:10.1371/journal.pntd.0007629)
Supplement: S1 Method — (DOCX) [file pntd.0007629.s001.docx]

**Explanatory variables for Amazonian model**

**Amazonian model**

Environmental variables

The Shuttle Radar Topography Mission (SRTM) elevation was obtained from <https://lpdaac.usgs.gov/products/srtmgl30v021/>.

Global Mammal Richness Grids represent the number of species for all 151 mammal families present in Amazonia, and are available at

<http://sedac.ciesin.columbia.edu/data/set/species-global-mammal-richness-2015> [1]. Mammal richness is used as a proxy of both habitat diversity and reservoir host biodiversity.

Vegetation biomass was used as a descriptor of forest typology and disturbance. It was created by the combination of two existing data sets of vegetation aboveground biomass (AGB) using an independent reference data set of field observations and locally calibrated high‐resolution biomass maps [2]. The data are available at <https://www.wur.nl/en/Research-Results/Chair-groups/Environmental-Sciences/Laboratory-of-Geo-information-Science-and-Remote-Sensing/Research/Integrated-land-monitoring/Forest_Biomass.htm>.

Forest canopy height is an indicator of species diversity and abundance of large mammal species [3]. Space-borne light detection and ranging (LiDAR) using 2005 data from the Geoscience Laser Altimeter System (GLAS) aboard ICESat (Ice, Cloud, and land Elevation Satellite) were used to map forest vertical structure globally [4]. The variable was downloaded at <https://www.nasa.gov/topics/earth/features/forest20120217.html>.

Bioclimatic variables

The 19 bioclimatic variables (WorldClim version 2) are average monthly climate data for annual trends from 1970 to 2000, such as annual mean temperature, isothermality, mean temperature in the warmest and coldest quarters, and mean precipitation in wettest and driest quarters [5]. The WorldClim climate was calculated by aggregation across a target temporal range of 1970–2000. Data were interpolated across regions with a low station density based on satellite data. All data were downloaded at WorldClim.org.

Anthropogenic and human-related variables

The Gridded Population of the World version 3 (GPW3) population density database projected for 2010 was used and accessed free online in the Socioeconomic Data and Applications Center (SEDAC) website. The core Global Rural–Urban Mapping Project Urban Extents surface used night-time light satellite imagery to differentiate urban areas [6]. GPW3 is a revision which updates the criteria for urban areas to those areas where population density is greater than or equal to 1,000 people per square kilometre.

For the poverty we used the G-Econ database (gecon.yale.edu) that consider economic data, at the smallest administrative division available, and spatially rescale these data to create a 1-km × 1-km gridded surface of the globe (Nordhaus, 2006, 2008). This rescaling estimates the gross cell product conceptually like gross domestic product, referring to the total market value of all final goods and services produced within 1 year. All gross cell product values were then adjusted using purchasing power parity in $US for the years 1990, 1995, 2000 and 2005 [7] and the mean was computed across all years for each gridded cell globally, which can be considered as an indicator of the overall standard of living and welfare within that area [7].

The global HFP data set of the last of the wild project (downloaded at <http://sedac.ciesin.columbia.edu/>) is the Human Influence Index (HII) normalised by biome and realm. The HII is a global data set of 1-km^2^ grid cells, created from eight global data layers covering human population density, human land use and infrastructure (built-up areas, night time lights, land use/land cover) and human access (coastlines, roads, railroads, navigable rivers). In Amazonia, HII values range from 0 to 64, with the zero value representing no human influence and 64 maximum human influence, using all eight measures of human presence. As a composite synthetic variable, we used HII as a proxy for human presence and its influence on ecosystems. The databases used to build the HII were the most complete and accurate data sources at the time. However, changes in population density, land transformation or accessibility in some Amazonian areas have occurred since HII construction (2009) and are not reflected in the current analysis.

**Explanatory variables for the French Guiana model**

Together with the bioclimatic variables [5] we also used another complementary set of variables, all recently developed at the French Guiana scale and consequently expected to provide a more precise view of the landscapes.

Cloud coverage was analysed with the spatial extent of fog/low stratus using the frequency distribution by means of night-time MODIS (Moderate Resolution Imaging Spectroradiometer) satellite data for the years 2007–2010 based on brightness temperature differences between the thermal and mid-infrared bands [8].

The HFP index for French Guiana sums up the expected and proven disturbances on biodiversity. The index was developed by superimposing geographical and human data, including human population density, land use, settlements and camps, mining and forest activities, tracks, roads and rivers [9], and was updated in 2012.

AGB estimates stem from a spatial predictive model [10]. This prediction map is based on the inventories of 2507 field plots in undisturbed rainforest (0.4–0.5 ha) distributed throughout French Guiana. The model was developed by kriging-regression to include spatial and environmental effects on AGB.

Canopy height was based on LiDAR waveform metrics extracted from the GLAS (Geoscience Laser Altimeter System) space-borne LiDAR data and terrain information derived from the SRTM, DEM (digital elevation model) or derived from principal component analysis (PCA) of GLAS waveforms [11]. An additional set of physical descriptors of the landscapes was hand-made for the present work, including the distance to river courses, the distance to the closest 500-m-high relief, the distance to the nearest forest edge, the percentage of the cells covered by high forest and the density of track and road networks.

Point distribution methodology

For the first method, at each occurrence point one of the following HFP classes was considered: HFP scoring lower than 29, ranging from 30 to 50, or greater than 51. For each of these three classes exclusion and distribution buffers of varying sizes were created: the lower the score, the smaller the exclusion and distribution buffers were (Table 2). For the Amazon model the exclusion buffers’ radii ranged from 0.5 to 7.5 km and for the French Guiana model the radii ranged from 0.5 to 2 km. Concerning the distribution buffers’ radii, they ranged from 3 to 10 km for the Amazon model and from 3 to 6 km for the French Guiana model. The distribution area, where the occurrence points were randomly distributed (data management tool, ArcTool Box, ArcGis 10.4) was represented by the surface of the distribution buffer minus the surface of the exclusion buffer. For the second method, we did not generate an automatised exclusion buffer and we did not distribute the cases in areas where the HFP was greater than 50 for the Amazon model and more than 40 for the model for French Guiana. The size of the distribution buffer remained the same as in method 1. For the third method, we combined the first two methods: the exclusion buffers’ radii were the same as in method 1 and within the distribution buffer we extended the exclusion area with all areas with a HFP value greater than 50 and 40 for the Amazon and French Guiana models, respectively. For the Amazon model, the size of the distribution buffers was extended and ranged from 3 to 22.5 km. We kept the same size for distribution buffers for the French Guiana model.

[1] Center For International Earth Science Information Network-CIESIN-Columbia University; NatureServe. Gridded Species Distribution: Global Mammal Richness Grids, 2015 Release 2015. doi:10.7927/H4N014G5.

[2] Avitabile V, Herold M, Heuvelink GBM, Lewis SL, Phillips OL, Asner GP, et al. An integrated pan-tropical biomass map using multiple reference datasets. Global Change Biology 2016;22:1406–20. doi:10.1111/gcb.13139.

[3] de Thoisy B de, Fayad I, Clément L, Barrioz S, Poirier E, Gond V. Predators, Prey and Habitat Structure: Can Key Conservation Areas and Early Signs of Population Collapse Be Detected in Neotropical Forests? PLOS ONE 2016;11:e0165362. doi:10.1371/journal.pone.0165362.

[4] Simard M, Pinto N, Fisher JB, Baccini A. Mapping forest canopy height globally with spaceborne lidar. Journal of Geophysical Research 2011;116. doi:10.1029/2011JG001708.

[5] Fick SE, Hijmans RJ. WorldClim 2: new 1‐km spatial resolution climate surfaces for global land areas. International Journal of Climatology 2017;37:4302–15. doi:10.1002/joc.5086.

[6] Center For International Earth Science Information Network-CIESIN-Columbia University; International Food Policy Research Institute-IFPRI; The World Bank; Centro Internacional De Agricultura Tropical-CIAT. Global Rural-Urban Mapping Project, Version 1 (GRUMPv1): Urban Extents Grid 2011. doi:10.7927/H4GH9FVG.

[7] Nordhaus WD. Geography and macroeconomics: New data and new findings. PNAS 2006;103:3510–7. doi:10.1073/pnas.0509842103.

[8] Obregon A, Gehrig-Downie C, Gradstein SR, Bendix J. The potential distribution of tropical lowland cloud forest as revealed by a novel MODIS-based fog/low stratus night-time detection scheme. Remote Sensing of Environment 2014;155:312–24. doi:10.1016/j.rse.2014.09.005.

[9] de Thoisy B, Richard-Hansen C, Goguillon B, Joubert P, Obstancias J, Winterton P, et al. Rapid evaluation of threats to biodiversity: human footprint score and large vertebrate species responses in French Guiana. Biodivers Conserv 2010;19:1567–84. doi:10.1007/s10531-010-9787-z.

[10] Guitet S, Hérault B, Molto Q, Brunaux O, Couteron P. Spatial Structure of Above-Ground Biomass Limits Accuracy of Carbon Mapping in Rainforest but Large Scale Forest Inventories Can Help to Overcome. PLOS ONE 2015;10:e0138456. doi:10.1371/journal.pone.0138456.

[11] Fayad I, Baghdadi N, Bailly J-S, Barbier N, Gond V, Hajj M, et al. Canopy Height Estimation in French Guiana with LiDAR ICESat/GLAS Data Using Principal Component Analysis and Random Forest Regressions. Remote Sensing 2014;6:11883–914. doi:10.3390/rs61211883.
