# Supplementary material for: Ecological niche modelling for predicting the risk of cutaneous leishmaniasis in the Neotropical moist forest biome
Source: PLoS Negl Trop Dis. 2019 Aug 14;13(8):e0007629. doi: 10.1371/journal.pntd.0007629 (PMC6693739; doi:10.1371/journal.pntd.0007629)

**Supplemental results:**

AUC and omission results for models performed without case occurrences in Colombia and French Guiana.

|  | Without Colombia | Without French Guiana |
| --- | --- | --- |
| AUC | 0.845 | 0.852 |
| Variables | (1) Pop_density (2) HFP (3) Bioclim4 (4) Mammals (5) Aboveground biomass | (1) HFP (2) Bioclim16 (3) Bioclim2 (4) Distance_c500m |
| Minimum training presence training omission | 0.0000 | 0.0000 |
| Minimum training presence test omission | 0.0017 | 0.0010 |
| 10 percentile training presence training omission | 0.0996 | 0.0999 |
| 10 percentile training presence test omission | 0.1039 | 0.1051 |

Risk map of CL in Amazonian basin without cases occurrence of Colombia.


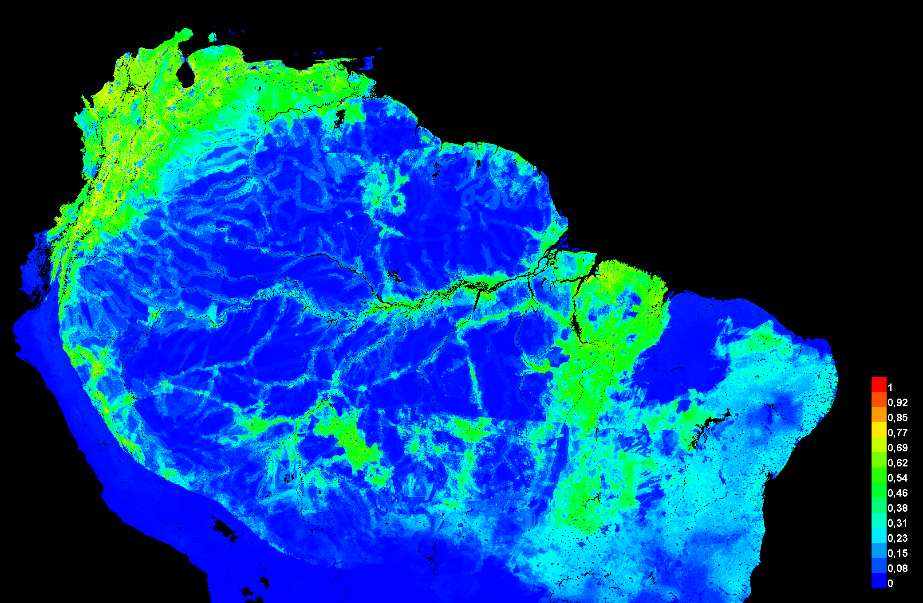


Risk map of CL in Amazonian basin without cases occurrence of French Guiana.


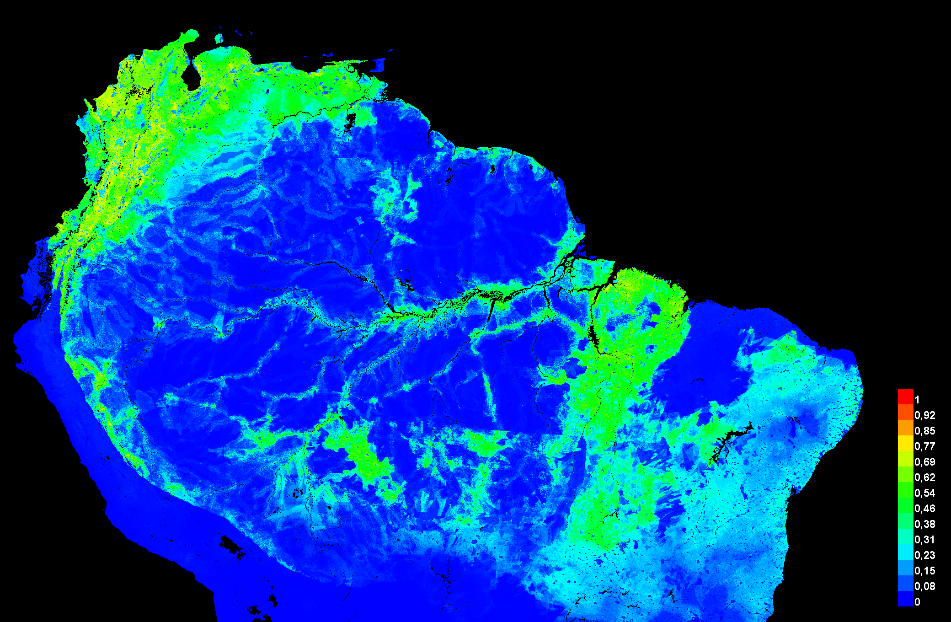

Supplement: S1 Results — (DOCX) [file pntd.0007629.s002.docx]
