# Supplementary figures and images for: Ecological niche modelling for predicting the risk of cutaneous leishmaniasis in the Neotropical moist forest biome
Source: PLoS Negl Trop Dis. 2019 Aug 14;13(8):e0007629. doi: 10.1371/journal.pntd.0007629 (PMC6693739; doi:10.1371/journal.pntd.0007629)

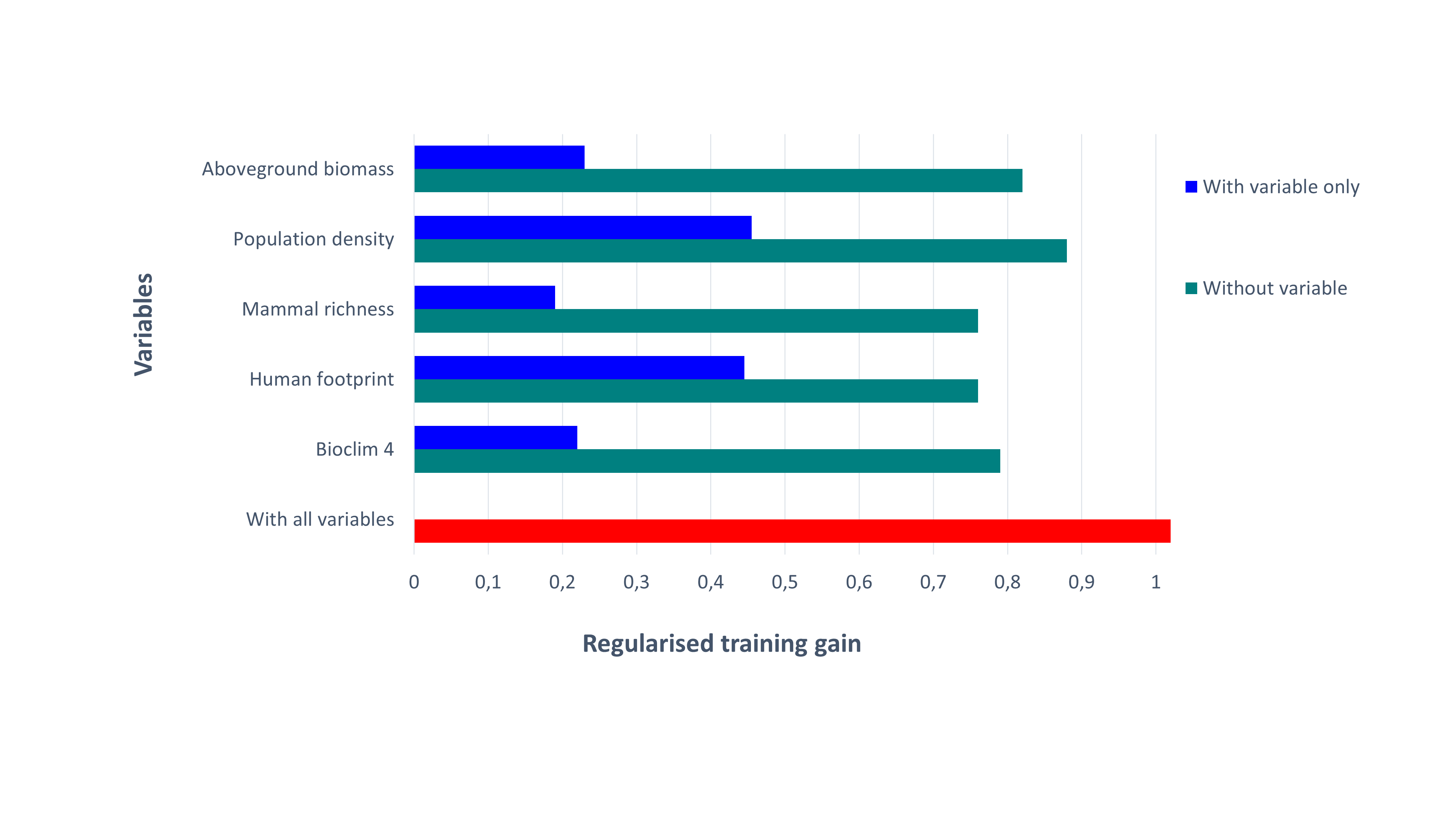

Supplement: S1 Fig — These tests represent the contribution of each variable independently of the others. Values shown are averages over replicate runs. (TIF) [file pntd.0007629.s004.tif]

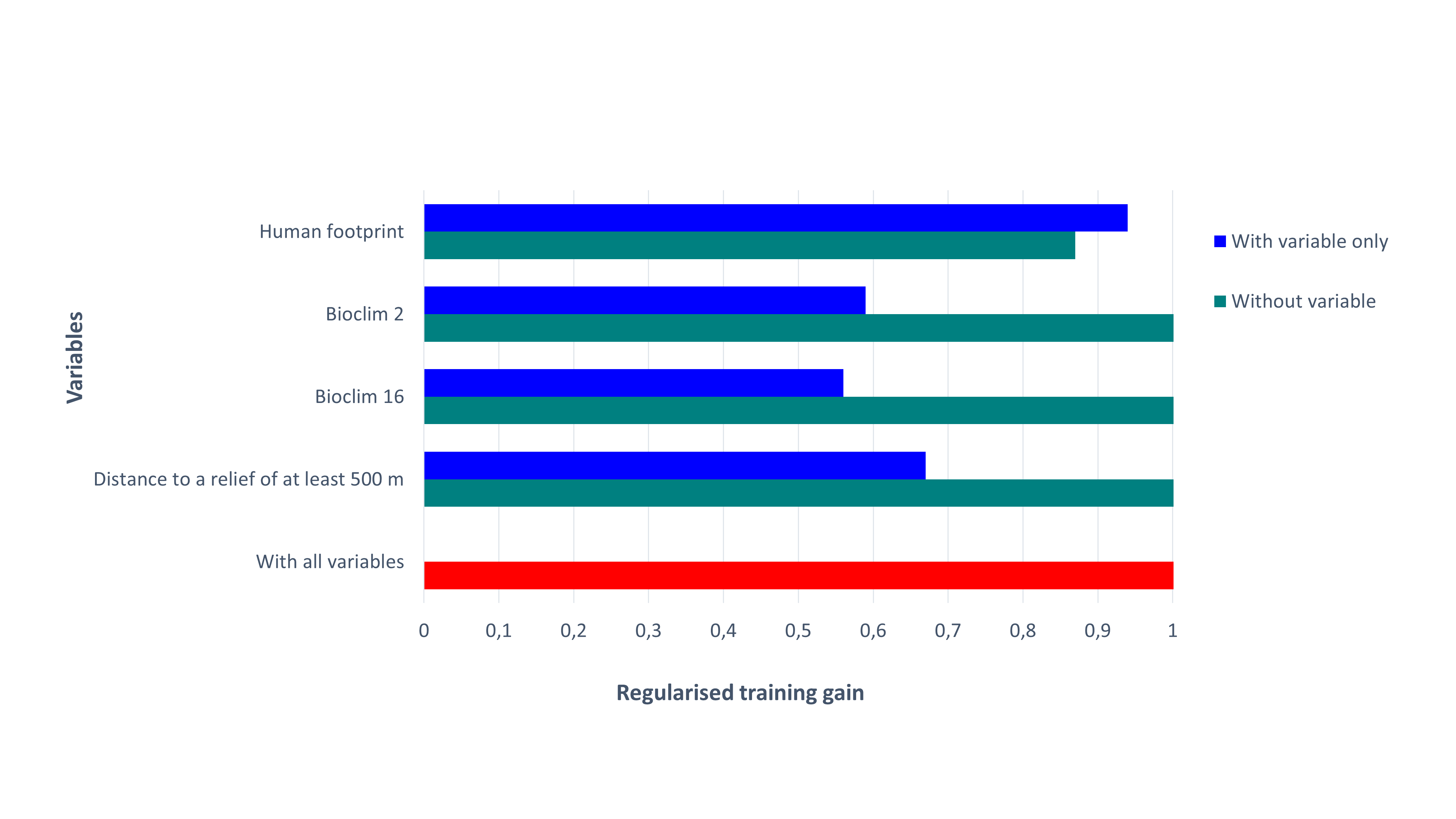

Supplement: S2 Fig — These tests represent the contribution of each variable independently of the others. (TIF) [file pntd.0007629.s005.tif]
